# Supplementary material for: The Prevalence and Clinical Impact of Transition Zone Anastomosis in Hirschsprung Disease: A Systematic Review and Meta-Analysis
Source: Children (Basel). 2023 Aug 30;10(9):1475. doi: 10.3390/children10091475 (PMC10528601; doi:10.3390/children10091475)
Supplement: Supplementary file 1 [file children-10-01475-s001.zip › children-2541341-supplementary.pdf]

children

MDPI

Supporting information

Table S1. Definitions and measurements for diagnosing TZA in each study.

Author	Definition TZ	Determination of aganglionic bowel during initial surgery	Determination TZA
Chatoorgoon, 2011 (1)	Ganglionic cells with hypertrophic nerves	NR	Full thickness biopsy
Coe, 2012 (2)	The presence of ganglion cells in the submucosa or myenteric plexus with hypertrophied and hyperplastic nerves.	NR	Biopsy
Dingemans, 2017 (3)	NR	NR	Full thickness biopsy
Farrugia, 2003 (4)	Diminished numbers of ganglion cells in the myenteric plexus and submucosa. Whole-mount preparations show increased irregularity and wider spacing of the polygonal network of ganglia and nerve fibers of the myenteric plexus. Occasional acetylcholinesterase staining hypertrophic bundles of extrinsic nerves also are seen.	Frozen section biopsy	Biopsy
Gad El-Hak, 2010 (5)	NR	NR	Postoperative pathology
Ghose, 2000 (6)	More subtle abnormalities of innervation resent between normal bowel and aganglionic bowel.	Frozen section biopsy	Further biopsies were not performed
Ghosh, 2017 (7)	Decreased number of ganglionic myenteric nervous plexuses.	Frozen section biopsy	Frozen section of the proximal margin
Gupta, 2019 (8)	NR	Frozen section biopsy	Absence of ganglion cells in the proximal part of the resected bowel
Hadidi, 2007 (9)	NR	Frozen section biopsy	Biopsy
Han, 2019 (10)	NR	Frozen section biopsy (not all patients)	Contrast study
Imvised, 2016 (11)	NR	Frozen section biopsy	Permanent histology report from resected bowel
Jiang, 2019 (12)	NR	Frozen section biopsy	Full thickness biopsy
Keshtgar, 2003 (13)	NR	NR	NR
Kobayashi, 1995 (14)	NR	Frozen section biopsy	NR
Langer, 2000 (15)	NR	Frozen section biopsy	NR
Langer, 2003 (16)	NR	Frozen section biopsy	NR
Langer, 2004 (17)	NR	NR	Rectal biopsy
Lawal, 2011 (18)	The presence of hypertrophic nerves in the submucosa, with normal	Frozen section biopsy	Rectal biopsy

	ganglion cells or absence of ganglion cells		
Peña, 2007 (19)	NR	NR	Rectal biopsy
Peng, 2020 (20)	NR	NR	NR
Pini-Prato, 2010 (21)	NR	NR	Rectal/full-thickness biopsy
Pini-Prato, 2020 (22)	NR	NR	NR
Polley, 1986 (23)	NR	Frozen section biopsy	NR
Ralls, 2014 (24)	NR	Frozen section biopsy	Biopsy
Schulten, 2000 (25)	NR	Frozen section biopsy	NR
Schweizer, 2007 (26)	Hypoganglionosis, dysganglionosis, aganglionosis, and a pattern of intestinal neuronal	NR	NR
Sheng, 2012 (27)	NR	Frozen section biopsy	Rectal biopsy
Stensrud, 2010 (28)	NR	Frozen section biopsy	NR
Van Leeuwen, 2000 (29)	NR	NR	Rectal biopsy
Vu, 2010 (30)	NR	Frozen section biopsy	NR
Weber, 1999 (31)	NR	NR	NR
Wilcox, 1998 (32)	NR	NR	Rectal biopsy
Wildhaber, 2004 (33)	NR	NR	Rectal/full-thickness biopsy and contrast enema
Xia, 2016 (34)	The presence of ganglion cells in the submucosa or myenteric plexus with hypertrophied and hyperplastic nerves.	Frozen section biopsy	Rectal biopsy

NR = not reported

Table S2. Indications for all redo pull-through procedures in this systematic review.

Indication redo PT	Number of patients
Transition zone anastomosis	323
Anastomotic stricture	94
Fistulae	31
Mega pouch	25
Chronic constipation	20
Adhesions	10
Intestinal neuronal dysplasia	9
Twisted anastomosis	8
Retraction of bowel	5
Dilated distal bowel segment	5

Narrowed rectal cuff	4
Recurrent Hirschsprung-associated enterocolitis	3
Anastomotic retraction	3
Pouchitis	2
Disruption of the anastomosis	2
Prescacral sinus	2
Bleeding	1
Necrosis	1
Severe fecal incontinence	1
Leak	1
Rectal prolapse	1
Rectal diverticula	1
Missing	*48*
**Total**	**600**

Figure S1. Funnel plot showing the risk of publication bias

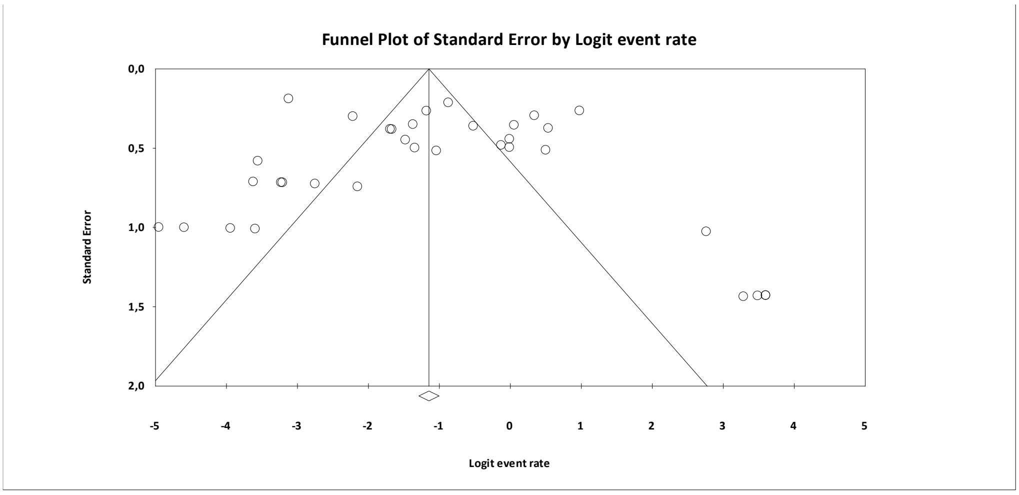

Alpha-level of 0.05 was considered statistically significant.
Egger's regression: t=0.486, p=0.711

Full Search strategy
Pubmed
("Hirschsprung Disease"[Mesh] OR hirschsprung*[tiab] OR congenital megacolon[tiab] OR aganglionic megacolon[tiab] OR rectosigmoid colon aganglionosis[tiab] OR rectosigmoid aganglionosis[tiab] OR congenital intestinal aganglionosis[tiab] OR colonic aganglionosis[tiab] OR total colonic aganglionosis[tiab] OR residual aganglionosis[tiab])
AND
("Anastomosis, Surgical"[Mesh] OR "Reoperation"[Mesh] OR "Hirschsprung Disease/surgery"[MAJR] OR anastomos*[tiab] OR redo surger*[tiab] OR pull through[tiab] OR pullthrough[tiab] OR reoperation*[tiab] OR re-operation*[tiab])
AND
("Pathology"[Mesh] OR "Biopsy"[Mesh] OR patholog*[tiab] OR cytopatholog*[tiab] OR histopatholog*[tiab] OR biops*[tiab] OR transition zone*[tiab])
EMBASE (Ovid):
Database(s): Embase Classic+Embase 1947 to 2019 February 07
Hirschsprung disease/ or (hirschsprung* or congenital megacolon or aganglionic megacolon or rectosigmoid colon aganglionosis or rectosigmoid aganglionosis or congenital intestinal aganglionosis or colonic aganglionosis or total colonic aganglionosis or residual aganglionosis).ti,ab,kw.
AND

exp anastomosis/ or reoperation/ or pull through operation/ or (anastomos* or redo surger* or redo pull-through or transition zone pull through or reoperation* or re-operation*).ti,ab,kw.
AND
exp pathology/ or exp biopsy/ or (patholog* or cytopatholog* or histopatholog* or biops* or transition zone*).ti,ab,kw.
Cinahl (Ebsco):
(MH "Hirschsprung Disease+") OR (TI (hirschsprung* or congenital megacolon or aganglionic megacolon or rectosigmoid colon aganglionosis or rectosigmoid aganglionosis or congenital intestinal aganglionosis or colonic aganglionosis or total colonic aganglionosis or residual aganglionosis) OR AB (hirschsprung* or congenital megacolon or aganglionic megacolon or rectosigmoid colon aganglionosis or rectosigmoid aganglionosis or congenital intestinal aganglionosis or colonic aganglionosis or total colonic aganglionosis or residual aganglionosis))
AND
(MH "Pathology+") OR (MH "Biopsy+") OR (TI (patholog* or cytopatholog* or histopatholog* or biops* or transition zone*) OR AB (patholog* or cytopatholog* or histopatholog* or biops* or transition zone*))
AND
(MH "Anastomosis, Surgical+") OR (MH "Reoperation+") OR (TI (anastomos* or redo surger* or redo pull-through or transition zone pull through or reoperation* or re-operation*) OR AB (anastomos* or redo surger* or redo pull-through or transition zone pull through or reoperation* or re-operation*))
Web of Science:
TOPIC: (hirschsprung* or congenital megacolon or aganglionic megacolon or rectosigmoid colon aganglionosis or rectosigmoid aganglionosis or congenital intestinal aganglionosis or colonic aganglionosis or total colonic aganglionosis or residual aganglionosis)
AND
TOPIC: (anastomos* or redo surger* or redo or pull through or reoperation* or re-operation*)
AND
TOPIC: (patholog* or cytopatholog* or histopatholog* or biops* or transition zone*

1. Chatoorgoon K, Pena A, Lawal TA, Levitt M. The problematic Duhamel pouch in Hirschsprung's disease: manifestations and treatment. Eur J Pediatr Surg. 2011;21(6):366-9.
2. Coe A, Collins MH, Lawal T, Louden E, Levitt MA, Pena A. Reoperation for Hirschsprung disease: pathology of the resected problematic distal pull-through. Pediatric and developmental pathology : the official journal of the Society for Pediatric Pathology and the Paediatric Pathology Society. 2012;15(1):30-8.
3. Dingemans A, van der Steeg H, Rassouli-Kirchmeier R, Linssen MW, van Rooij I, de Blaauw I. Redo pull-through surgery in Hirschsprung disease: Short-term clinical outcome. J Pediatr Surg. 2017;52(9):1446-50.
4. Farrugia MK, Alexander N, Clarke S, Nash R, Nicholls EA, Holmes K. Does transitional zone pull-through in Hirschsprung's disease imply a poor prognosis? J Pediatr Surg. 2003;38(12):1766-9.
5. Gad El-Hak NA, El-Hemaly MM, Negm EH, El-Hanafy EA, Abdel Messeh MH, Abdel Bary HH. Functional outcome after Swenson's operation for Hirshsprung's disease. Saudi journal of gastroenterology : official journal of the Saudi Gastroenterology Association. 2010;16(1):30-4.
6. Ghose SI, Squire BR, Stringer MD, Batcup G, Crabbe DCG. Hirschsprung's disease: Problems with transition-zone pull-through. Journal of Pediatric Surgery. 2000;35(12):1805-9.
7. Ghosh DN, Liu YR, Cass DT, Soundappan SSV. Transition zone pull-through in Hirschsprung's disease: a tertiary hospital experience. Anz Journal of Surgery. 2017;87(10):780-3.
8. Gupta DK, Khanna K, Sharma S. Experience with the Redo Pull-Through for Hirschsprung's Disease. Journal of Indian Association of Pediatric Surgeons. 2019;24(1):45-51.
9. Hadidi A, Bartoli F, Waag KL. Role of transanal endorectal pull-through in complicated Hirschsprung's disease: experience in 18 patients. J Pediatr Surg. 2007;42(3):544-8.

10. Han JW, Youn JK, Oh C, Kim HY, Jung SE, Park KW. Why Do the Patients with Hirschsprung Disease Get Redo Pull-Through Operation? Eur J Pediatr Surg. 2018.

11. Imvised T. Multicenter Experience of Primary Transanal Endorectal Pull-Through Operation in Childhood Hirschsprung's Disease. In: Vejchapipat P, editor. Journal of the medical association of Thailans since 19182016.

12. Jiang M, Li CL, Cao GQ, Tang ST. Laparoscopic Redo Pull-Through for Hirschsprung Disease Due to Innervation Disorders. J Laparoendosc Adv S. 2019;29(3):424-9.

13. Keshtgar AS, Ward HC, Clayden GS, de Sousa NM. Investigations for incontinence and constipation after surgery for Hirschsprung's disease in children. Pediatr Surg Int. 2003;19(1-2):4-8.

14. Kobayashi H, Hirakawa H, Surana R, O'Briain DS, Puri P. Intestinal neuronal dysplasia is a possible cause of persistent bowel symptoms after pull-through operation for Hirschsprung's disease. J Pediatr Surg. 1995;30(2):253-7; discussion 7-9.

15. Langer JC, Seifert M, Minkes RK. One-stage Soave pull-through for Hirschsprung's disease: a comparison of the transanal and open approaches. J Pediatr Surg. 2000;35(6):820-2.

16. Langer JC, Durrant AC, de la Torre L, Teitelbaum DH, Minkes RK, Caty MG, et al. One-stage transanal Soave pullthrough for Hirschsprung disease: a multicenter experience with 141 children. Ann Surg. 2003;238(4):569-83; discussion 83-5.

17. Langer JC. Persistent obstructive symptoms after surgery for Hirschsprung's disease: development of a diagnostic and therapeutic algorithm. J Pediatr Surg. 2004;39(10):1458-62.

18. Lawal TA, Chatoorgoon K, Collins MH, Coe A, Pena A, Levitt MA. Redo pull-through in Hirschprung's disease for obstructive symptoms due to residual aganglionosis and transition zone bowel. Journal of Pediatric Surgery. 2011;46(2):342-7.

19. Pena A, Elicevik M, Levitt MA. Reoperations in Hirschsprung disease. J Pediatr Surg. 2007;42(6):1008-13; discussion 13-4.

20. Peng C, Chen Y, Pang W, Zhang T, Wang Z, Wu D, et al. Redo Transanal Soave Pull Through with or without Assistance in Hirschsprung Disease: An Experience in 46 Patients. Eur J Pediatr Surg. 2021;31(2):182-6.

21. Pini-Prato A, Mattioli G, Giunta C, Avanzini S, Magillo P, Bisio GM, et al. Redo surgery in Hirschsprung disease: what did we learn? Unicentric experience on 70 patients. J Pediatr Surg. 2010;45(4):747-54.

22. Pini Prato A, Arnoldi R, Faticato MG, Mariani N, Dusio MP, Felici E, et al. Minimally Invasive Redo Pull-Throughs in Hirschsprung Disease. J Laparoendosc Adv Surg Tech A. 2020;30(9):1023-8.

23. Theodore Z. Polley J. The definitive management of Hirschsprung's disease with the endorectal pull-through procedure. Pediatric Surgery International · June 19861986.

24. Ralls MW, Freeman JJ, Rabah R, Coran AG, Ehrlich PF, Hirschl RB, et al. Redo pullthrough for Hirschsprung disease: a single surgical group's experience. J Pediatr Surg. 2014;49(9):1394-9.

25. Schulten D, Holschneider AM, Meier-Ruge W. Proximal segment histology of resected bowel in Hirschsprung's disease predicts postoperative bowel function. Eur J Pediatr Surg. 2000;10(6):378-81.

26. Schweizer P, Berger S, Schweizer M, Holschneider AM, Beck O. Repeated pull-through surgery for complicated Hirschsprung's disease--principles derived from clinical experience. J Pediatr Surg. 2007;42(3):536-43.

27. Sheng Q, Lv Z, Xiao X. Re-operation for Hirschsprung's disease: experience in 24 patients from China. Pediatr Surg Int. 2012;28(5):501-6.

28. Stensrud KJ, Emblem R, Bjornland K. Functional outcome after operation for Hirschsprung disease--transanal vs transabdominal approach. J Pediatr Surg. 2010;45(8):1640-4.

29. van Leeuwen K, Teitelbaum DH, Elhalaby EA, Coran AG. Long-term follow-up of redo pull-through procedures for Hirschsprung's disease: efficacy of the endorectal pull-through. J Pediatr Surg. 2000;35(6):829-33; discussion 33-4.
30. Vu PA, Thien HH, Hiep PN. Transanal one-stage endorectal pull-through for Hirschsprung disease: experiences with 51 newborn patients. Pediatr Surg Int. 2010;26(6):589-92.
31. Weber TR, Fortuna RS, Silen ML, Dillon PA. Reoperation for Hirschsprung's disease. J Pediatr Surg. 1999;34(1):153-6; discussion 6-7.
32. Wilcox DT, Kiely EM. Repeat pull-through for Hirschsprung's disease. J Pediatr Surg. 1998;33(10):1507-9.
33. Wildhaber BE, Pakarinen M, Rintala RJ, Coran AG, Teitelbaum DH. Posterior myotomy/myectomy for persistent stooling problems in Hirschsprung's disease. J Pediatr Surg. 2004;39(6):920-6; discussion -6.
34. Xia X, Li N, Wei J, Zhang W, Yu D, Zhu T, et al. Laparoscopy-assisted versus transabdominal reoperation in Hirschprung's disease for residual aganglionosis and transition zone pathology after transanal pull-through. J Pediatr Surg. 2016;51(4):577-81.
